# Comprehensive Genomic Profiling of Rare Tumors in China: Routes to Immunotherapy

**DOI:** 10.3389/fimmu.2021.631483

**Published:** 2021-02-25

**Authors:** Shuhang Wang, Yuan Fang, Ning Jiang, Shujun Xing, Qin Li, Rongrong Chen, Xin Yi, Zhiqian Zhang, Ning Li

**Affiliations:** ^1^ Key Laboratory of Carcinogenesis and Translational Research (Ministry of Education), Department of Cell Biology, Peking University Cancer Hospital and Institute, Beijing, China; ^2^ Clinical Cancer Center, National Cancer Center/National Clinical Research Center for Cancer/Cancer Hospital, Chinese Academy of Medical Sciences and Peking Union Medical College, Beijing, China; ^3^ Department of Medical Center, Geneplus-Beijing Institute, Beijing, China

**Keywords:** rare tumors, immunotherapy, PD-L1, TMB, HLA-I

## Abstract

Treatment options for rare tumors are limited, and comprehensive genomic profiling may provide useful information for novel treatment strategies and improving outcomes. The aim of this study is to explore the treatment opportunities of patients with rare tumors using immune checkpoint inhibitors (ICIs) that have already been approved for routine treatment of common tumors. We collected immunotherapy-related indicators data from a total of 852 rare tumor patients from across China, including 136 programmed cell death ligand-1 (PD-L1) expression, 821 tumors mutational burden (TMB), 705 microsatellite instability (MSI) and 355 human leukocyte antigen class I (HLA-I) heterozygosity reports. We calculated the positive rates of these indicators and analyzed the consistency relationship between TMB and PD-L1, TMB and MSI, and HLA-I and PD-L1. The prevalence of PD-L1 positive, TMB-H, MSI-, and HLA-I -heterozygous was 47.8%, 15.5%, 7.4%, and 78.9%, respectively. The consistency ratio of TMB and PD-L1, TMB and MSI, and HLA-I and PD-L1 was 54.8% (78/135), 87.3% (598/685), and 47.4% (54/114), respectively. The prevalence of the four indicators varied widely across tumors systems and subtypes. The probability that neuroendocrine tumors (NETs) and biliary tumors may benefit from immunotherapy is high, since the proportion of TMB-H is as high as 50% and 25.4% respectively. The rates of PD-L1 positivity, TMB-H and MSI-H in carcinoma of unknown primary (CUP) were relatively high, while the rates of TMB-H and MSI-H in soft tissue tumors were both relatively low. Our study revealed the distribution of immunotherapeutic indicators in patients with rare tumors in China. Comprehensive genomic profiling may offer novel therapeutic modalities for patients with rare tumors to solve the dilemma of limited treatment options.

## Introduction

Currently, there exists no consensus definition for the category of “rare tumors,” either worldwide or in China. Because of the low incidence rate, it is difficult to carry out large-scale studies on these diseases. Due to this lack of study, patients with rare tumors are often unable to take advantage of therapeutic advances. In China, there is a lack of research on rare tumors, leading to limited options for effective treatment and poor survival and prognosis for these patients compared to those with common tumors.

Based on the definition of rare tumors by Food and Drug Administration (FDA), National Cancer Institute and European Society for Medical Oncology (1, 2), Professor Li Ning’s team from Clinical Trial Canter, National Cancer Hospital, Chinese Academy of Medical Sciences and Peking Union Medical College, proposed the definition of rare tumors in China first time. This definition was based on data from the National Cancer Registration Office of China National Cancer Canter, combined with the incidence rate of cancer, the characteristics of the population in China, classification according to the International Classification of Diseases and the OncoTrees (http://oncotree.mskcc.org/). The incidence threshold for a “rare tumor” was initially set at 2.5/100,000. In a previous study, we compared the incidence of therapeutic targets in rare tumors in the cBioPortal database (https://www.cbioportal.org/datasets) and a Chinese population database (Geneplus database). We found that the incidence of therapeutic targets in rare tumors in the Chinese population was significantly higher than in the general population (53.43% vs. 20.40% respectively). Moreover, in the Chinese population, prevalence of targetable genomic alterations within those rare tumors (ALK, BRAF, BRCA2, CDKN2A, EGFR, HER2, KIT, MET, ROS1) was 32.4%, which is more than 3 times that which is found in the general population according to cBioPortal (3).

Using the National Comprehensive Cancer Network and Chinese Society of Clinical Oncology guidelines as the main data sources (https://www.nccn.org, http://www.csco.org.cn), we collected records for the tumor types that fit the current definition of “rare tumors,” and investigated the availability and efficacy of various treatment modalities. With respect to targeted therapy, of more than 100 rare tumor subtypes, only 16 tumor types were involved in targeted therapy studies, but the disease control rate and objective response rate of rare tumors with targetable mutations are better than those treated with standard treatment. With respect to immunotherapy, of more than 100 rare tumor subtypes, the research on immunotherapy involved less than 17 tumor types. Some curative effect has been preliminarily observed, but only skin squamous cell carcinoma has been approved by the FDA as an indication for Libtayo (PD-1, cemiplimab-rwlc). These results suggest that even in the context of scarcity of clinical trials and guidelines for diagnosis and treatment, there are still some rare tumors included in these studies, which has yielded promising preliminary results for targeted therapy and immunotherapy.

Immunotherapy is revolutionary cancer treatment. Programmed cell death protein-1 and programmed cell death ligand-1 (PD-L1) checkpoint inhibitors can benefit a variety of malignant tumors patients, which has been shown in many studies (4–6). PD-L1 overexpression (7, 8), mismatch repair deficiency (dMMR) (9–11), microsatellite instability-high status (MSI-H) (10–12), or high tumor mutational burden (TMB-H) (13–15) are the main predictive molecular biomarkers in these studies. Human leukocyte antigen class I (HLA-I) is a prognostic biomarker of great concern, representing the impact of host germline genetics on immune checkpoint inhibitors (ICIs) therapies response. CD8 + T cells have been shown to be the main factor in the antitumor activity of ICIs, and the peptide presentation process on the cell surface depends on HLA-I (16, 17). More diverse tumor antigens presented to T cells can benefit from heterozygous HLA-I genotypes (18). Some studies support that patients with HLA-I heterozygosity, had longer overall survival (OS) in pan-cancers (17), while others show that it wasn’t the case in non-small-cell lung cancer (NSCLC) (19).

Within rare tumors, some reports have shown that immunotherapy has demonstrated the efficacy in some subtypes, including biliary tumors, neuroendocrine tumors (NETs), and carcinoma of unknown primary (CUP), among others (20–23). The same predictive molecular biomarkers that are used for common cancers (described above) were used in these studies (20, 22, 23), and whether HLA-I heterozygosity improves OS is still unknown.

The purpose of this study was to analyze the prevalence of the immunotherapy-related indicators described above within rare tumors in China, so as to provide more insight into the treatment options for these patients.

## Methods

### Patient Recruitment

According to the definition and update of rare tumors published/established by the China National Cancer Center (3), we collected and retrospectively analyzed data on immunotherapy-related indicators from a total of 852 rare tumors patients in the Geneplus database, including 136 reports of PD-L1 expression, 821 reports of TMB, 705 of MSI and 355 of HLA-I heterozygosity.

The patients were enrolled from multiple medical canters and hospitals in China from September 2015 to February 2020. After signed written informed consent, all patients were tested by next generation sequencing (NGS) in Geneplus-Beijing Institute. Meanwhile, all patients were stratified into different clinicopathological groups according to the OncoTrees. During data analysis, two subtype tumors namely biliary tumors (including gallbladder cancer and extrahepatic cholangiocarcinoma) and NETs drew our attentions due to the high prevalence of TMB. **(** **Supplementary Table 1**)

### PD-L1 Expression

PD-L1 expression was assessed in formalin fixed paraffin embedded (FFPE) tumor tissues using the PD-L1 IHC 22C3 pharmDx assay (Dako, Carpinteria, CA, USA) in 94 patients; using the SP263 pharmDx assay (Ventana Automated Systems, Inc., Tucson, AZ, USA) in 21 patients; and using an unknown method in 21 patients (The PD-L1 test results of these patients were obtained from the previous medical records, and the detection method was not described).

The 22C3 pharmDx assay were performed according to the manufacturers’ instructions. The sections were stained with the anti-PD-L1 22C3 mouse monoclonal primary antibody, and then the EnVision FLEX visualization system (Agilent, Santa Clara, CA, USA) was performed on an Autostainer Link 48 system (Dako). The negative control reagents and cell line were also tested simultaneously as control (24).

For SP263 pharm Dx assay, OptiView DAB IHC Detection kit (Ventana Medical Systems, Basel, Switzerland) was used to stain the sections with SP263 anti-PD-L1 rabbit monoclonal primary antibody, and the analysis was performed on Ventana Bench-Mark XT automated staining platform (Ventana Automated Systems).

The results of PD-L1 immunohistochemistry (IHC) were interpreted by pathologists. The expression of PD-L1 in both tumor cells and immune cells was evaluated. The criterion of PD-L1 positive staining in tumor cells was that the complete or partial circumferential linear membrane staining can be distinguished from background and diffuse cytoplasmic staining at any intensity (25). After recording the proportion of positive cells on the whole section, the PD-L1 positive rate of tumor cells was scored relative to the whole tumor area (26). PD-L1 expression in tumor infiltrating lymphocytes was defined as any staining intensity in cell membrane or cytoplasm. The threshold of PD-L1 positive was 1%.

### Next-Generation Sequencing

All tissue samples included in this study were reexamined pathologically to confirm the histological classification and to ensure that at least 20% of the tumor cells were present for adequate detection. Genomic profiling was performed by Gene+Seq 2000 instrument or Illumina Nextseq CN 500 in the Geneplus-Beijing laboratory, which was accredited by American College of Pathologists (27, 28). Briefly, QIAamp DNA FFPE Tissue kit (Qiagen, Valencia, CA) was used to extract genomic tumor DNA from serial sections of FFPE tumor tissues. ctDNA was isolated from 4 to 5mL of plasma using the QIAamp Circulating Nucleic Acid Kit (Qiagen, Valencia, CA). DNA from leukocytes was extracted using the DNeasy Blood Kit (Qiagen, Valencia, CA). Sequencing libraries were prepared from ctDNA using KAPA DNA Library Preparation Kits (Kapa Biosystems, Wilmington, MA, USA), and genomic DNA sequencing libraries were prepared with Illumina TruSeq DNA Library Preparation Kits (Illumina, San Diego, CA). Libraries were hybridized to custom-designed biotinylated oligonucleotide probes (Roche NimbleGen, Madison, WI, USA) targeting 1,021 genes (~1.4 Mbp genomic regions of 1,021 cancer-related genes) **(** **Supplementary Table 2**) and HLA-I locus (A, B, and C). Prepared libraries were sequenced on using the Illumina Nextseq CN 500 (Illumina, San Diego, CA) or Gene+Seq 2000 (Geneplus-Beijing, China). Target capture sequencing required a minimal mean effective depth of coverage of 100× in leukocytes, 300× in tumor tissue and 1,000× in cell-free DNA samples.

Sequencing data were analyzed using default parameters. After removing adaptor sequences and low-quality reads, Burrows-Wheeler Aligner (BWA; version 0.7.12-r1039) was used to aligned the clean reads to the reference human genome (hg19). GATK (version 3.4-46-gbc02625) was performed for realignment and recalibration. MuTect (version 1.1.4) and NChot were used for single nucleotide variants (SNV) calling (29). GATK and CONTRA (v2.0.8) were performed to identify small inserts and deletions (InDels), and somatic copy number alternations, respectively. Finally, Integrative Genomics Viewer was used to manually verified all of the final candidate variants.

### Biomarker Analysis

#### TMB Analysis

Somatic nonsynonymous SNV and InDels mutations in coding regions, with allele frequency ≥ 0.03 in tumor tissue sample or ≥ 0.005 in ctDNA sample respective, were included in TMB calculation. TMB was defined as the number of above mutations per megabase of genome. Based on 2000 samples from Geneplus database, the threshold of TMB-H was identified as the top quartile and determined to be ≥ 9 mutations per megabase (30, 31).

#### MSI Status

MSIsensor (v0.2) was used to inferred the MSI statuses, which reported the percentage of somatic unstable microsatellites in predefined microsatellite regions in our panel based on chi-squared test (32). All parameters used the default settings. According to the MSIsensor scores of tumor samples and matched normal samples, the MSI-H threshold was established by MSI polymerase chain reaction (PCR) and MMR IHC cross validation. And the threshold of MSI-H was 8.

#### HLA-I Typing

HLA-I typing was done using the OptiType v1.0 to obtain the four-digit HLA type at each locus of a patient (33). OptiType performs HLA typing using a combinatorial optimization approach. Reads were mapped to a reference panel consisting of HLA Class I allele sequences centered around their most polymorphic, and functionally most important region, exons 2 and 3 (34). HLA I-homozygous was defined as homozygosity for at least one HLA-I locus (A, B, or C), and HLA I- heterozygous as heterozygosity for all of the three HLA-I locus.

## Results

### Clinicopathological Characteristics of Patients

Eight hundred and fifty-two patients (852) with rare tumors were included in this study. **Table 1** summarized the clinicopathological characteristics of all patients. The median age was 54, and male patients accounted for 53.6% (457/852). Among these patients, 671, 160, 10, 10, and 1 patients respectively used tumor tissue, ctDNA, pleural effusion, peritoneal effusion, as well as cerebrospinal fluid (CSF) samples for genetic analysis. These 852 cases included 91 tumor subtypes in rare tumor types, with neural, soft tissue, CUP, digestive, and respiratory systems as the top 5 tumors systems including 264, 180, 113, 98, and 78 patients, respectively.

**Table 1:** Clinicopathological characteristics of patients.

Characteristic	Pts. (N=852) (%)
Age, years	
median	54
range	1–91
Gender	
female	395(46.4%)
male	457(53.6%)
Specimen.	
tumor tissue	671(78.8%)
ctDNA	160(18.8%)
pleural effusion	10(1.2%)
peritoneal effusion	10(1.2%)
CSF	1(0.1%)
System	
1.head and neck	33(3.9%)
2.digestive	98(11.5%)
3.respiratory	78(9.2%)
4.reproductive	31(3.6%)
5.urinary	6(0.7%)
6.multiple system	25(2.9%)
7.skin	16(1.9%)
8.soft tissue	180(21.1%)
9.bone	6(0.7%)
10.endocrine	2(0.2%)
11.neural	260(30.5%)
12.CUP	117(13.7%)

### Predictive Factors

Within the 136 patients who underwent PD-L1 immunohistochemistry, 65 patients had PD-L1 positive tumors (47.8%). CUP, respiratory, multiple system, digestive and soft tissue systems were the top 5 systems with 76.5% (13/17), 65.4% (17/26), 44.4% (4/9), 40.0% (8/20), and 39.4% (13/33) positivity rates, respectively.

**Figure 1:**
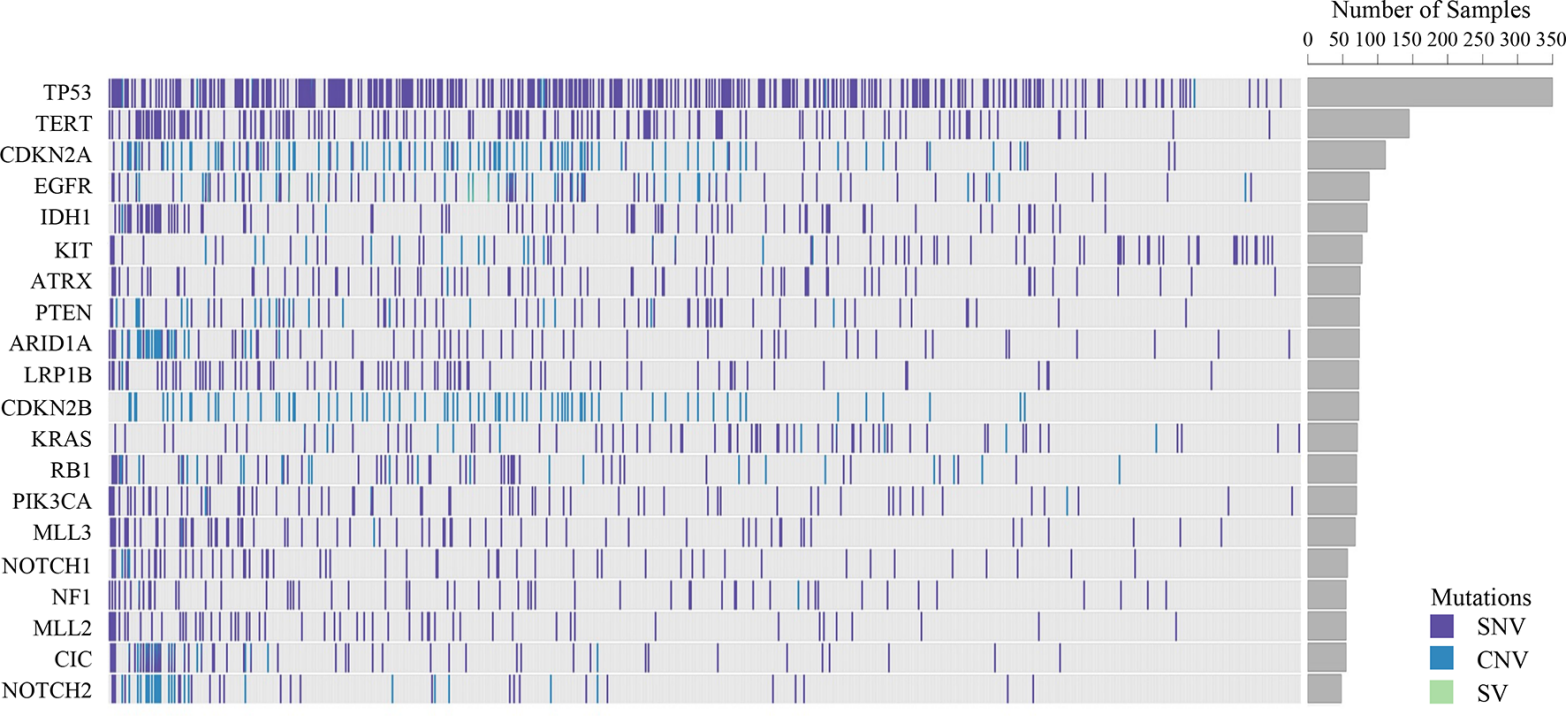
Top 20 mutant genes of all samples.

**Figure 2:**
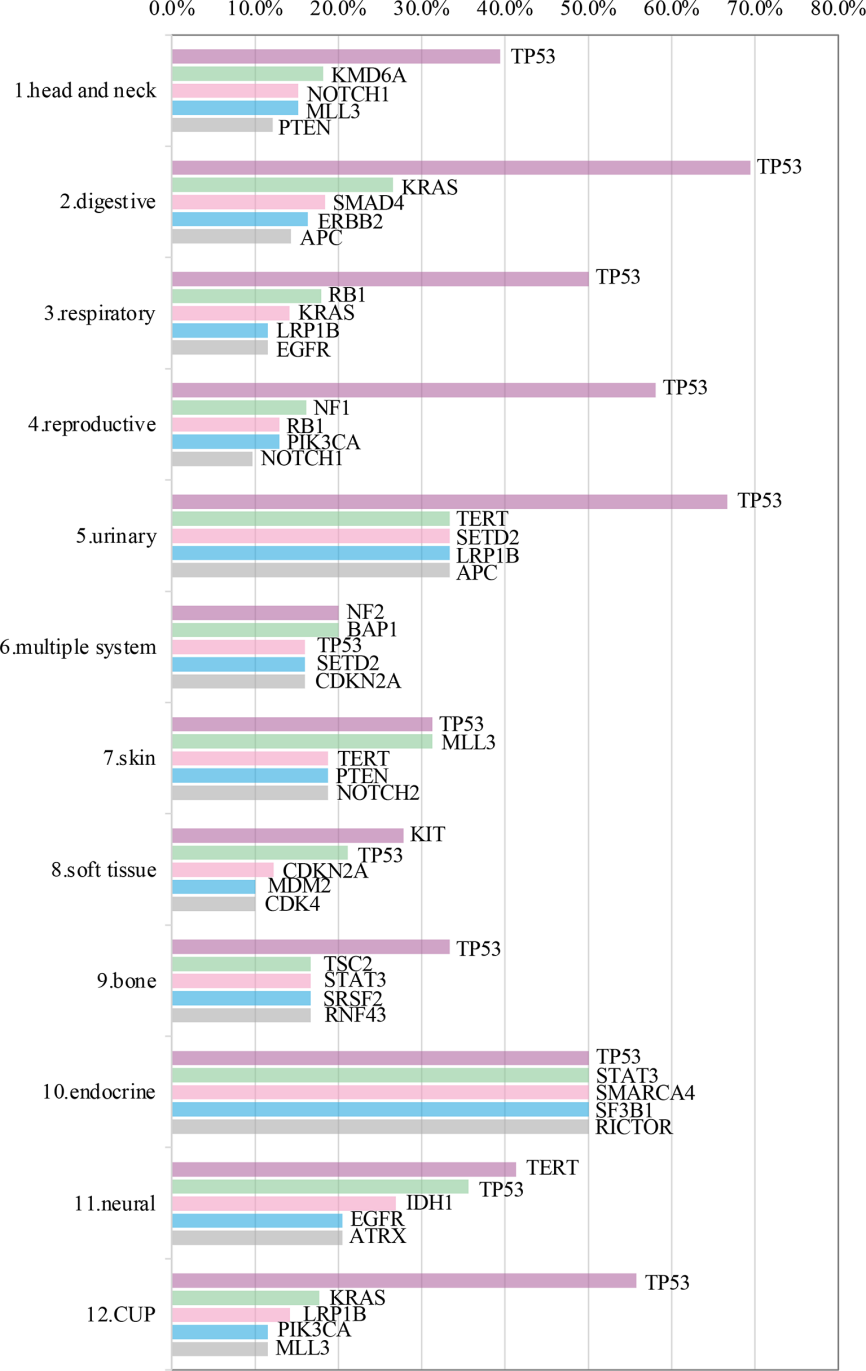
Top 5 mutant genes in 12 systems.

Somatic mutations were detected in most patients (98.9%, 843/852). The most common mutant genes were TP53 (40.8%), TERT (17.2%), and CNKN2A (13.4%) (Top 20 mutant genes were summarized in **Figure 1**). Except NF2, KIT and TERT were the most common mutant genes in multiple system, soft tissue system and neural system, respectively, TP53 was the most common mutant gene in the other nine systems (Top 5 mutant genes in 12 systems were summarized in **Figure 2**).

TMB-H was identified in 127 patients among 821 patients (15.5%). Prevalence of TMB-H varied widely across tumor systems, ranging from 0% in patients with bone system disease to 50.0% in patients in urinary or endocrine system disease. Urinary, endocrine, respiratory, skin and CUP systems were the top 5 systems with 50.0% (3/6), 50.0% (1/2), 27.8% (20/72), 26.7% (4/15), and 21.2% (24/113) TMB-H rate, respectively. Considering the tumor subtypes, NETs and biliary tumors were both higher, reaching 50% (9/18) and 25.4% (15/59) respectively.

MSI-H was identified in 7.4% patients (52/705). Bone, neural, respiratory, reproductive and head and neck systems were the top 5 systems with 25.0% (1/4), 15.6% (39/250), 5.0% (3/60), 5.0% (1/20), and 4.8% (1/21) positivity rates, respectively.

**Figure 3:**
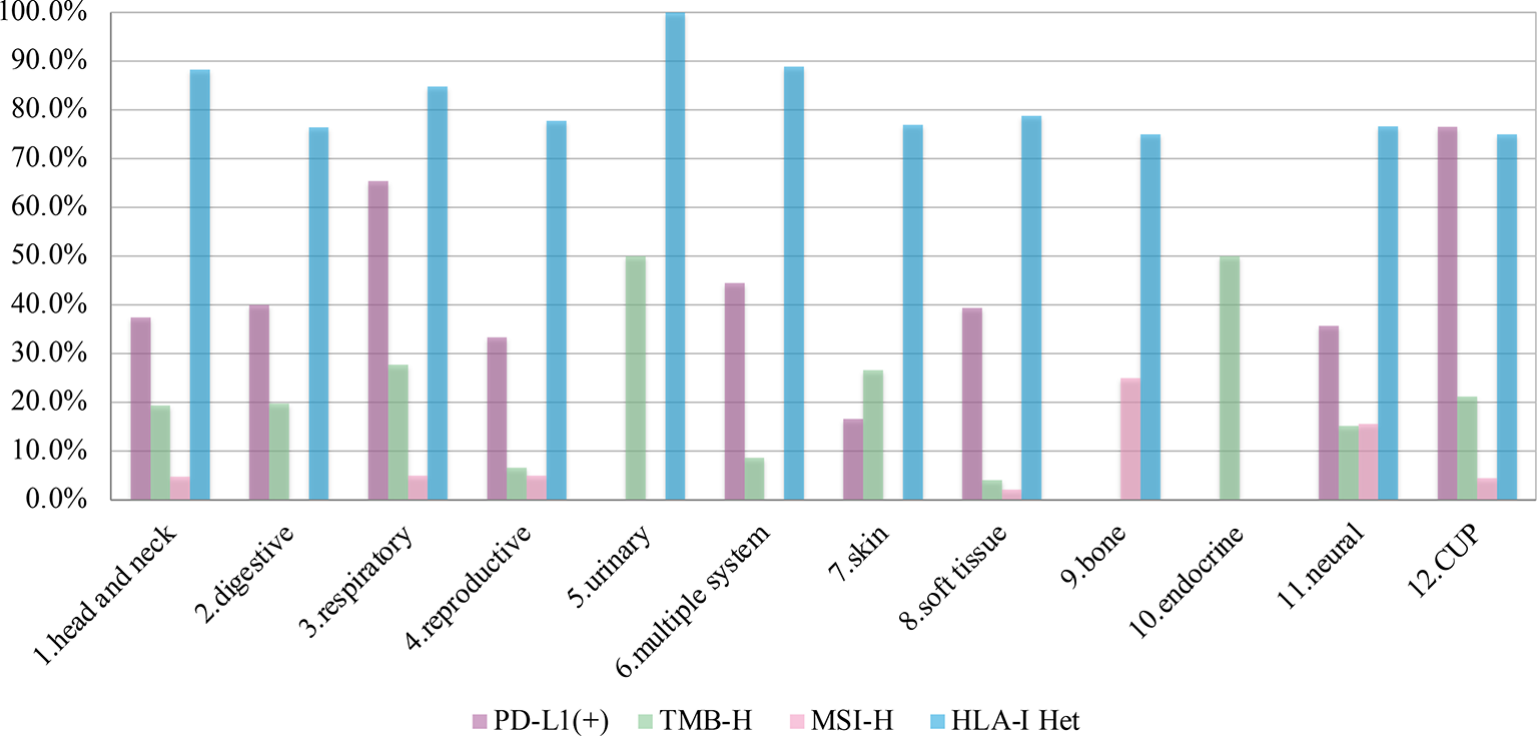
The prevalence of programmed cell death ligand-1 (PD-L1) positive, tumors mutational burden (TMB)-H, microsatellite instability (MSI)-H, and human leukocyte antigen class I (HLA)-I Het in 12 systems.

It should be noted that the rates of PD-L1 positivity, TMB-H, and MSI-H in CUP were relatively high, with 76.5% (13/17), 21.1% (24/113), and 4.5% (4/89), respectively. While the proportion of both TMB-H and MSI-H in soft tissue sarcomas was very low, with 4.1% (7/171) and 2.1% (3/143) respectively. (Prevalence of immunotherapy related indicators in rare tumor samples are summarized in **Table 2** and **Figure 3**).

**Table 2:** Prevalence of immunotherapy related indicators in rare tumor samples.

Systems	PD-L1 test	PD-L1(+)	%	22C3 test	22C3(+)	%	SP263 test	SP263(+)	%	TMB test	TMB-H	%	MSI test	MSI-H	%	HLA-I	HLA-I Het	%
Total	136	65	47.8	94	38	40.4	21	18	85.7	821	127	15.5	705	52	7.4	355	280	78.9
1.head and neck	8	3	37.5	5	1	20.0	1	1	100.0	31	6	19.4	21	1	4.8	17	15	88.2
2.digestive	20	8	40.0	13	3	23.1	6	5	83.3	96	19	19.8	78	0	0.0	68	52	76.5
biliary	11	5	45.5	7	2	28.6	3	3	100.0	59	15	25.4	51	0	0.0	40	29	72.5
3.respiratory	26	17	65.4	13	9	69.2	5	5	100.0	72	20	27.8	60	3	5.0	33	28	84.8
neuroendocrine	6	2	33.3	2	1	50.0	1	1	100.0	18	9	50.0	17	1	5.9	6	5	83.3
4.reproductive	3	1	33.3	2	1	50.0				30	2	6.7	20	1	5.0	9	7	77.8
5.urinary										6	3	50.0	6	0	0.0	2	2	100.0
6.multiple system	9	4	44.4	7	3	42.9	1	0	0.0	23	2	8.7	20	0	0.0	18	16	88.9
7.skin	6	1	16.7	4	0	0.0	2	1	50.0	15	4	26.7	13	0	0.0	13	10	76.9
8.soft tissue	33	13	39.4	24	7	29.2	4	4	100.0	171	7	4.1	143	3	2.1	80	63	78.8
9.bone										5	0	0.0	4	1	25.0	4	3	75.0
10.endocrine										2	1	50.0	1	0	0.0			
11.neural	14	5	35.7	12	4	33.3				257	39	15.2	250	39	15.6	47	36	76.6
12.CUP	17	13	76.5	14	10	71.4	2	2	100.0	113	24	21.2	89	4	4.5	64	48	75.0

**Figure 4:**
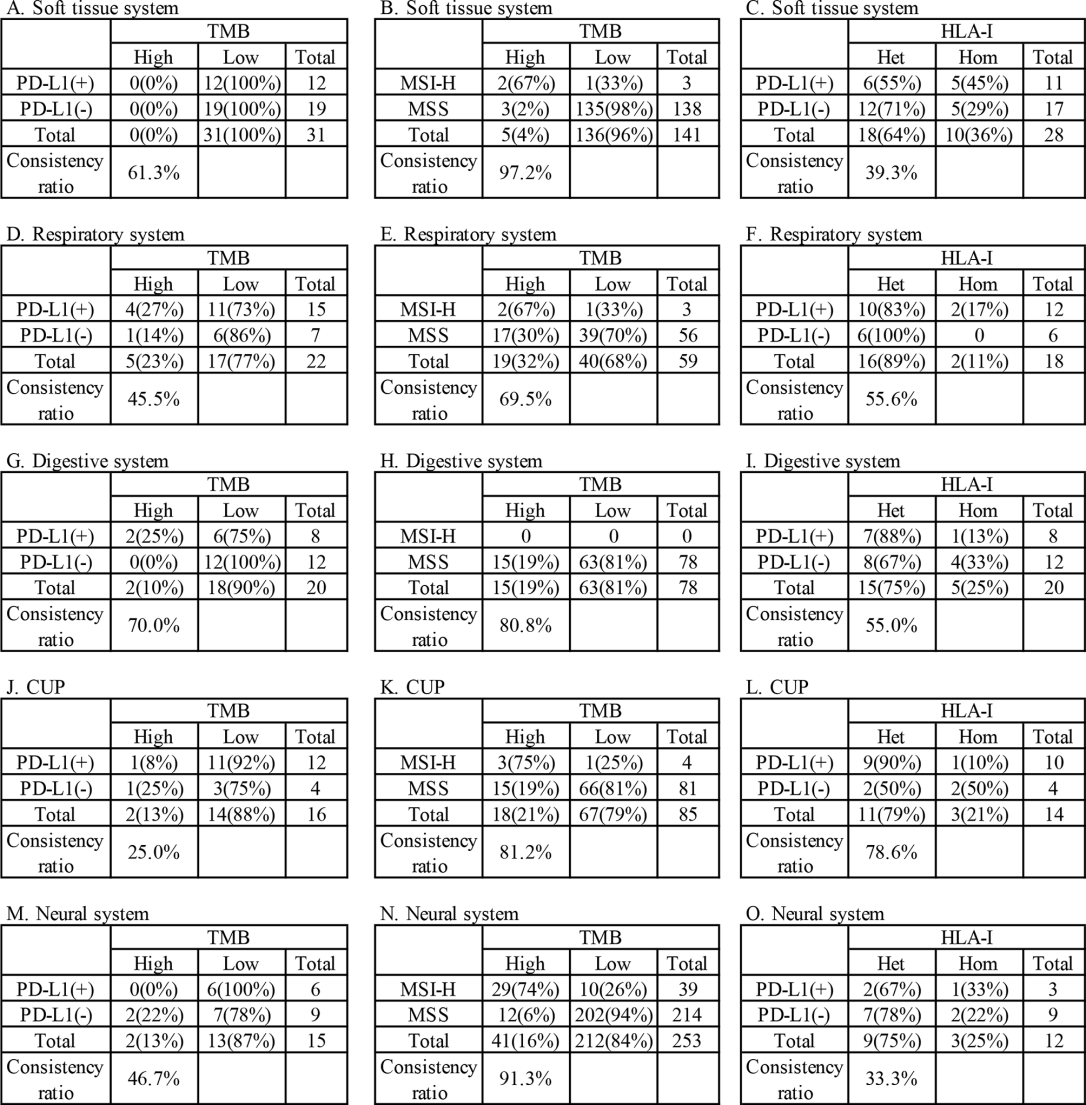
Consistency analysis of tumors mutational burden (TMB) and human leukocyte antigen class I (HLA-I) with programmed cell death ligand-1 (PD-L1) and microsatellite instability (MSI) in main systems. **(A–C)** soft tissue systems, **(D–F)** respiratory system, **(G-I)** digestive system, **(J–L)** CUP, **(M–O)** neural system.

Among the above patients, 135 patients were tested for both TMB and PD-L1, while 685 patients were tested for both TMB and MSI. The consistency ratio of TMB results and PD-L1 results was 54.8% (78/135), while that of TMB and MSI was 87.3% (598/685) (**Table 3**). We summarized the consistency data of five systems with larger sample size, including soft tissue, respiratory, digestive, CUP, and neural system. The consistency data of most systems were consistent with the overall consistency data, but there were some special cases in some systems, including the consistency ratio of TMB and PD-L1 in digestive system was as high as 70.0% (14/20), and that of TMB and MSI in respiratory system was as low as 69.5% (41/59) (**Figure 4**).

**Table 3:** Consistency analysis of tumors mutational burden (TMB) and human leukocyte antigen class I (HLA-I) with programmed cell death ligand-1 (PD-L1) and microsatellite instability (MSI).

Testing method	TMB (tissue)		TMB (ctDNA)		HLA-I	
	High	Low	High	Low	Het	Hom
PD-L1 (+)	8	50	1	6	40	12
PD-L1 (−)	4	60	1	5	48	14
MSI-H	37	14	0	0		
MSS	59	533	14	28		

### Prognostic Factors

A total of 78.9% (280/355) patients were identified as HLA class I-heterozygous. The top 5 systems were urinary, multiple system, head and neck, respiratory and soft tissue system with 100% (2/2), 88.9% (16/18), 88.2% (15/17), 84.8% (28/33), and 78.8% (63/80) heterozygous rate, respectively (**Figure 3**). Among them, 114 patients were tested for PD-L1, and the consistency ratio of HLA-I results and PD-L1 results was 47.4% (54/114) (**Table 3**). The consistency of the five systems with larger sample size were also summarized, and the consistency ratio of HLA-I and PD-L1 in CUP was as high as 78.6% (11/14) (**Figure 4**).

## Discussion

The purpose of this study is to explore potential novel indications for the treatment of rare tumors in China. Results show that the clinical benefit-related indicators for immunotherapy are frequently present in rare tumors, though their prevalence varied widely across tumor systems and subtypes.

**Figure 5:**
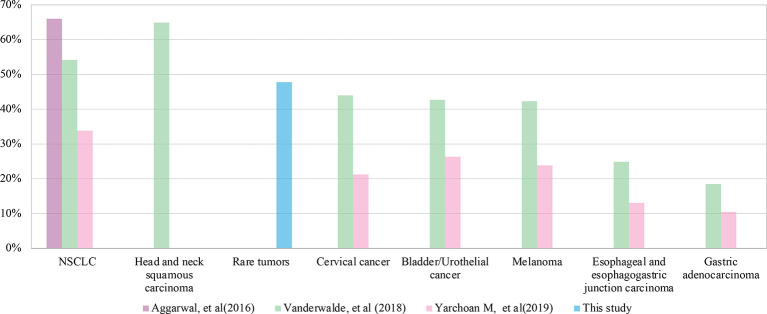
Comparison of programmed cell death ligand-1 (PD-L1) positive rates between rare tumors and common tumors in different studies.

PD-L1 is the first internationally recognized therapeutic indicator in immunotherapy. PD-L1 positivity is required in some indications approved for immunotherapy, including in NSCLC, gastric cancer, esophageal cancer, cervical cancer, head and neck tumor and triple negative breast cancer. We compared the prevalence of PD-L1 positivity in this study (47.8%) to those of several common cancers with approved indications of immunotherapy (**Figure 5**) (35–37). We found that the overall prevalence of PD-L1 positive in this study was higher than that of the above approved common tumors, except NSCLC (54.2%~66%) and head and neck tumor (64.9%). This suggests that rare tumors have a greater chance to benefit from immunotherapy than most common tumors. In addition to advanced tumors, studies are also underway to assess the predictive value of PD-L1 expression for early-stage tumors. In a neoadjuvant study of NSCLC, major pathologic response was found to be positively correlated with PD-L1 expression. In patients who have never given anti-tumor therapy, if pathological remission can be proved to be related to PD-L1 expression, other interference factors that lead to the heterogeneity of tumor PD-L1 detection are excluded (38). The predictive value of PD-L1 in early-stage rare tumors is another interesting area to explore.

TMB is another promising immunotherapeutic biomarker. Many studies have found that high TMB in immunotherapy is highly correlated with clinical benefit. For example, TMB-H in tissue (defined as >200 mutations in exome) was associated with durable clinical benefit and longer progression-free survival in NSCLC patients treated with pembrolizumab as monotherapy. Similarly, in patients with melanoma given ipilimumab, higher TMB in tissue (evaluated by whole-exome sequencing and measured as a continuous variable) was also associated with improved outcomes (39, 40). Additionally, in NSCLC patients treated with nivolumab combined with ipilimumab, at least 10 mutations per megabase of tissue TMB were associated with improved clinical outcomes (41, 42). It was also observed that in NSCLC patients treated with durvalumab plus tremelimumab or atezolizumab, TMB with ≥16 mutations per megabase in ctDNA based on blood samples was associated with improved clinical outcomes (43, 44). Data of some small retrospective studies also showed that issue TMB was associated with improved outcomes in ICIs for multiple tumor types (45, 46), other studies including the prospective KEYNOTE-158 study suggested that, across multiple tumor types patients with ICIs therapy, increased levels of tissue TMB were associated with higher response rates (20, 47).

However, some studies have shown that TMB cannot predict the efficacy of immunotherapy. Several studies, including KEYNOTE-021 and KEYNOTE-189, have shown that TMB cannot predict the clinical outcomes of corresponding first-line immunotherapy for NSCLC (48, 49). The overall prevalence of TMB-H in this study was 15.5%, similar to that reported in the KEYNOTE-158 study. Also, in our study, NETs and biliary tumors had much higher TMB-H rates than that in the KEYNOTE-158 study (43.8% vs 29.3%, 25.5% vs 4.0%, respectively) (20, 50). Given the high prevalence of TMB-H status in rare tumors, the effect of TMB on immunotherapy response in rare tumors deserves further exploration.

MSI status, along with PD-L1 and TMB, is another possibly independent, predictive indication for ICIs. MSI-H has been confirmed in many studies to predict the response of various solid tumors to ICIs and has been approved by FDA as the first indication biomarker for pan-cancer immunotherapy (9, 51). MSI is most common in colon and endometrial cancer (highly associated with Lynch syndrome), where it can be as high as 15% and 28% respectively, but relatively low in other cancers (52, 53). According to several large-scale studies, the overall incidence of MSI-H in all cancers is about 3% (36, 51, 54). In these studies, in addition to colon and endometrial cancer, the incidence of MSI-H in gastric adenocarcinoma (3.4%~9%) and small intestinal malignancies (4.6%~8%) is also relatively high, while it is low in NSCLC (<1%) and melanoma (nearly 0). In our analysis, the prevalence of MSI-H in rare tumors in China was 7.4%, which was higher than that reported across all cancers. Additionally, prevalence of MSI-H in head and neck, CUP and soft tissue systems tumors in this study was still higher than that reported in previous studies abroad (4.8% vs. 0.5%, 4.5% vs. 1.9%, 2.1% vs. 0.2%, respectively) (54).

This study also included HLA-I heterozygosity as a prognostic indicator of immunotherapy and was the first study on HLA-I heterozygosity in rare tumors. Previous studies have shown that in ICIs treatment patients across multiple cancer types (including NSCLC and melanoma), heterozygous HLA-I genotyps improved OS compared with patients who were homozygous for at least one HLA locus (17). Our data show a heterozygous rate of HLA-I of 78.9% in rare tumors, which was similar to that previously reported in NSCLC (77.5%~78.4%) (19).

In our analysis of the relationship between the indicators, the concordance between TMB and PD-L1 was only 54.8%, indicating they are independent in predicting the benefit of immunotherapy, which is the same as that of common tumors (55). The same situation was found in HLA-I and PD-L1, with a consistency of 47.4%. However, TMB and MSI showed a high positive correlation (87.3%), which was similar to that of colorectal cancers (36). The consistency data of most systems were consistent with the overall consistency data, but some systems shown particularity, which reminds us that further research can be put into these tumors.

Within the subgroups of rare tumors, we noted that the positivity rates of PD-L1, TMB-H and MSI-H in CUP were relatively high, indicating that immunotherapy is a worthwhile treatment option. The proportion of both TMB-H and MSI-H in soft tissue sarcomas is very low, suggesting that patients with such tumors are less likely to benefit from immunotherapy.

Due to the small number of cases in each rare tumor subtype, it is difficult to compare the details of each tumor subtype in this study. So we classified tumor subtypes into various tumor systems, and then compared the indicators. In addition, since the patients of some rare tumor systems were limited, especially in the urinary, bone and endocrine systems, the prevalence of the four indicators analyzed in this study may be divergent from the actual situation. However, this study captures the overall situation of immunotherapy-related indicators of rare tumors and supports that a considerable proportion of patients with rare tumors can benefit from immunotherapy.

Based on the previous study (CITE) and this study, we designed the PLATFORM study. PLATFORM is an open, non-randomized, multicohort, single arm, single center phase II clinical study in advanced rare solid tumors that have been treated with or without standard treatment. The main purpose of the PLATFORM study is to evaluate the safety and efficacy of targeted drugs approved in China and to evaluate/test targeted therapy for specific tumor driver genes in patients with advanced rare solid tumor patients who have corresponding targets, as well as to evaluate the safety and efficacy of ICIs (PD-1 antibodies) in patients with advanced rare solid tumors who have no druggable target mutations. Patients with advanced rare solid tumors who failed or did not have standard treatment will be included in the study. Based on the results of gene detection, the subjects carrying the targets “EGFR mutation, ALK gene fusion, ROS-1 gene fusion, MET gene amplification or mutation, BRAF mutation, BRCA1/2 mutation, HER-2 positive, KIT mutation and CDKN2A mutation” will be divided into 13 arms according to the types of gene variation, and will be divided into 9 targeted treatment study groups and given the corresponding targeted drug/agent (Almonertinib, Dacomitinib, Alectinib, Crizotinib, Vemurafenib, Niraparib, Pyrotinib, Imatinib, Palbociclib). Subjects without the above targets will be enrolled in the immunotherapy group and treated with PD-1 inhibitor monotherapy. During the treatment, the usage and dosage of the above drugs, the principle of dose adjustment and matters needing attention will all be referred to the drug labels and instructions. All AE/SAE of the above drugs in advanced rare solid tumors will be collected for safety analysis. After the patients are enrolled in the corresponding targeted treatment group, they will be treated according to the standard dosage/manufacturer’s recommended dosage until the disease progresses or intolerable adverse reactions occur. The PLATFORM study is the first platform study for rare tumors in the world. We look forward to increasing opportunities for Chinese patients with rare tumors to benefit from targeted therapy and immunotherapy through this world leading research method and innovative structure/design. (NCT04423185)

The most important purpose of this study is to raise awareness of the necessity of rare tumor research among Chinese clinical workers, government officials and drug investigators around the world. Even though there is no consensus and effective treatment guidelines in China, we think that promoting the development of new drugs and treatment strategies of rare tumors will be fruitful. In view of the high prevalence of immunotherapy related indicators in the rare tumors population and limited treatment options of these patients, adequate efforts should be made for rare tumors in the near future.

## Conclusions

This study included 852 tumor samples from patients whose tumors met the definition of rare tumor in China. We analyzed the prevalence of immunotherapy predictors and prognostic indicators, including PD-L1, TMB, MSI, and HLA-I, and their consistency. The results showed that a considerable proportion of rare tumor patients are positive for the above indicators, and especially that nearly half of patients were PD-L1 positive, suggesting that they could benefit from immunotherapy. Comprehensive genomic profiling may offer novel therapeutic modalities for patients with rare tumors to solve the dilemma of limited treatment options. All of the above facilitates the development of new drug investigations and treatment improvement for rare tumors in the future.

## Data Availability Statement

The datasets presented in this study can be found in online repositories. The names of the repository/repositories and accession number(s) can be found in the article/**Supplementary Material**.

## Author Contributions

NL designed the study. ZZ directed the data analysis and gave important suggestion on the revision. XY helped design the study but was not involved in the data analysis and revision. SW performed the study and analyzed the data. SW, YF, NJ, SX, QL, RC, and NL composed the manuscript. All authors contributed to the article and approved the submitted version.

## Conflict of Interest

The authors declare that the research was conducted in the absence of any commercial or financial relationships that could be construed as a potential conflict of interest.

## Supplementary Material

Supplementary Table 1 Patients’ clinicopathological subgroups and immunotherapy related indicators data.

Supplementary Table 2 1021 genes list.

## Abbreviations

CSF, cerebrospinal fluid; CUP, carcinoma of unknown primary; dMMR, mismatch repair-deficient; FDA, Food and Drug Administration; FFPE, formalin-fixed paraffin-embedded; HLA-I, human leukocyte antigen class I; ICIs, immune checkpoint inhibitors; IHC, immunohistochemistry; InDels, Small insertions and deletions; MSI, microsatellite instability; NETs, neuroendocrine tumors; NGS, next generation sequencing; NSCLC, non-small-cell lung cancer; OS, overall survival; PCR, polymerase chain reaction; PD-L1, programmed cell death ligand-1; SNV, single nucleotide variants; TMB, tumor mutational burden.

## References

[1] Gatta G van der Zwan JM Casali PG Siesling S Dei Tos AP Kunkler I. Rare cancers are not so rare: the rare cancer burden in Europe. Eur J Cancer (2011) 47(17):2493–511. 10.1016/j.ejca.2011.08.008 22033323

[2] Greenlee RT Goodman MT Lynch CF Platz CE Havener LA Howe HL. The occurrence of rare cancers in U.S. adults, 1995-2004. Public Health Rep (2010) 125(1):28–43. 10.1177/003335491012500106 20402194 PMC2789814

[3] Wang S Chen R Tang Y Yu Y Fang Y Huang H. Comprehensive Genomic Profiling of Rare Tumors: Routes to Targeted Therapies. Front Oncol (2020) 10:536. 10.3389/fonc.2020.00536 32373528 PMC7186305

[4] Darvin P Toor SM Sasidharan Nair V Elkord E. Immune checkpoint inhibitors: recent progress and potential biomarkers. Exp Mol Med (2018) 50(12):1–11. 10.1038/s12276-018-0191-1 PMC6292890 30546008

[5] Ribas A Wolchok JD. Cancer immunotherapy using checkpoint blockade. Science (2018) 359(6382):1350–5. 10.1126/science.aar4060 PMC7391259 29567705

[6] Akinleye A Rasool Z. Immune checkpoint inhibitors of PD-L1 as cancer therapeutics. J Hematol Oncol (2019) 12(1):92. 10.1186/s13045-019-0779-5 31488176 PMC6729004

[7] Patel SP Kurzrock R. PD-L1 Expression as a Predictive Biomarker in Cancer Immunotherapy. Mol Cancer Ther (2015) 14(4):847–56. 10.1158/1535-7163.MCT-14-0983 25695955

[8] Mok TSK Wu YL Kudaba I Kowalski DM Cho BC Turna HZ. Pembrolizumab versus chemotherapy for previously untreated, PD-L1-expressing, locally advanced or metastatic non-small-cell lung cancer (KEYNOTE-042): a randomised, open-label, controlled, phase 3 trial. Lancet (2019) 393(10183):1819–30. 10.1016/S0140-6736(18)32409-7 30955977

[9] Le DT Uram JN Wang H Bartlett BR Kemberling H Eyring AD. PD-1 Blockade in Tumors with Mismatch-Repair Deficiency. N Engl J Med (2015) 372(26):2509–20. 10.1056/NEJMoa1500596 PMC4481136 26028255

[10] Overman MJ McDermott R Leach JL Lonardi S Lenz HJ Morse MA. Nivolumab in patients with metastatic DNA mismatch repair-deficient or microsatellite instability-high colorectal cancer (CheckMate 142): an open-label, multicentre, phase 2 study. Lancet Oncol (2017) 18(9):1182–91. 10.1016/S1470-2045(17)30422-9 PMC6207072 28734759

[11] Le DT Kim TW Van Cutsem E Geva R Jager D Hara H. Phase II Open-Label Study of Pembrolizumab in Treatment-Refractory, Microsatellite Instability-High/Mismatch Repair-Deficient Metastatic Colorectal Cancer: KEYNOTE-164. J Clin Oncol (2020) 38(1):11–9. 10.1200/JCO.19.02107 PMC7031958 31725351

[12] Marcus L Lemery SJ Keegan P Pazdur R. FDA Approval Summary: Pembrolizumab for the Treatment of Microsatellite Instability-High Solid Tumors. Clin Cancer Res (2019) 25(13):3753–8. 10.1158/1078-0432.CCR-18-4070 30787022

[13] Samstein RM Lee CH Shoushtari AN Hellmann MD Shen R Janjigian YY. Tumor mutational load predicts survival after immunotherapy across multiple cancer types. Nat Genet (2019) 51(2):202–6. 10.1038/s41588-018-0312-8 PMC6365097 30643254

[14] Reck M Schenker M Lee KH Provencio M Nishio M Lesniewski-Kmak K. Nivolumab plus ipilimumab versus chemotherapy as first-line treatment in advanced non-small-cell lung cancer with high tumour mutational burden: patient-reported outcomes results from the randomised, open-label, phase III CheckMate 227 trial. Eur J Cancer (2019) 116:137–47. 10.1016/j.ejca.2019.05.008 31195357

[15] Jiang T Shi J Dong Z Hou L Zhao C Li X. Genomic landscape and its correlations with tumor mutational burden, PD-L1 expression, and immune cells infiltration in Chinese lung squamous cell carcinoma. J Hematol Oncol (2019) 12(1):75. 10.1186/s13045-019-0762-1 31299995 PMC6625041

[16] Gubin MM Zhang X Schuster H Caron E Ward JP Noguchi T. Checkpoint blockade cancer immunotherapy targets tumour-specific mutant antigens. Nature (2014) 515(7528):577–81. 10.1038/nature13988 PMC4279952 25428507

[17] Chowell D Morris LGT Grigg CM Weber JK Samstein RM Makarov V. Patient HLA class I genotype influences cancer response to checkpoint blockade immunotherapy. Science (2018) 359(6375):582–7. 10.1126/science.aao4572 PMC6057471 29217585

[18] Chowell D Krishna C Pierini F Makarov V Rizvi NA Kuo F. Evolutionary divergence of HLA class I genotype impacts efficacy of cancer immunotherapy. Nat Med (2019) 25(11):1715–20. 10.1038/s41591-019-0639-4 PMC7938381 31700181

[19] Negrao MV Lam VK Reuben A Rubin ML Landry LL Roarty EB. PD-L1 Expression, Tumor Mutational Burden, and Cancer Gene Mutations Are Stronger Predictors of Benefit from Immune Checkpoint Blockade than HLA Class I Genotype in Non-Small Cell Lung Cancer. J Thorac Oncol (2019) 14(6):1021–31. 10.1016/j.jtho.2019.02.008 30780001

[20] Marabelle A Fakih M Lopez J Shah M Shapira-Frommer R Nakagawa K. Association of tumour mutational burden with outcomes in patients with advanced solid tumours treated with pembrolizumab: prospective biomarker analysis of the multicohort, open-label, phase 2 KEYNOTE-158 study. Lancet Oncol (2020) 21(10):1353–65. 10.1016/S1470-2045(20)30445-9 32919526

[21] Naing A Meric-Bernstam F Stephen B Karp DD Hajjar J Rodon Ahnert J. Phase 2 study of pembrolizumab in patients with advanced rare cancers. J Immunother Cancer (2020) 8(1):e000347. 10.1136/jitc-2019-000347 32188704 PMC7078933

[22] D’Angelo SP Russell J Lebbe C Chmielowski B Gambichler T Grob JJ. Efficacy and Safety of First-line Avelumab Treatment in Patients With Stage IV Metastatic Merkel Cell Carcinoma: A Preplanned Interim Analysis of a Clinical Trial. JAMA Oncol (2018) 4(9):e180077. 10.1001/jamaoncol.2018.0077 29566106 PMC5885245

[23] Mehnert JM Panda A Zhong H Hirshfield K Damare S Lane K. Immune activation and response to pembrolizumab in POLE-mutant endometrial cancer. J Clin Invest (2016) 126(6):2334–40. 10.1172/JCI84940 PMC4887167 27159395

[24] Kim H Kwon HJ Park SY Park E Chung JH. PD-L1 immunohistochemical assays for assessment of therapeutic strategies involving immune checkpoint inhibitors in non-small cell lung cancer: a comparative study. Oncotarget (2017) 8(58):98524–32. 10.18632/oncotarget.21567 PMC5716747 29228707

[25] Phillips T Simmons P Inzunza HD Cogswell J Novotny J Jr Taylor C. Development of an automated PD-L1 immunohistochemistry (IHC) assay for non-small cell lung cancer. Appl Immunohistochem Mol Morphol (2015) 23(8):541–9. 10.1097/PAI.0000000000000256 PMC4561627 26317305

[26] Roach C Zhang N Corigliano E Jansson M Toland G Ponto G. Development of a Companion Diagnostic PD-L1 Immunohistochemistry Assay for Pembrolizumab Therapy in Non-Small-cell Lung Cancer. Appl Immunohistochem Mol Morphol (2016) 24(6):392–7. 10.1097/PAI.0000000000000408 PMC4957959 27333219

[27] Sun S Liu Y Eisfeld AK Zhen F Jin S Gao W. Identification of Germline Mismatch Repair Gene Mutations in Lung Cancer Patients With Paired Tumor-Normal Next Generation Sequencing: A Retrospective Study. Front Oncol (2019) 9:550. 10.3389/fonc.2019.00550 31297337 PMC6607931

[28] Zhuo M Guan Y Yang X Hong L Wang Y Li Z. The Prognostic and Therapeutic Role of Genomic Subtyping by Sequencing Tumor or Cell-Free DNA in Pulmonary Large-Cell Neuroendocrine Carcinoma. Clin Cancer Res (2020) 26(4):892–901. 10.1158/1078-0432.CCR-19-0556 31694833 PMC7024651

[29] Yang X Chu Y Zhang R Han Y Zhang L Fu Y. Technical Validation of a Next-Generation Sequencing Assay for Detecting Clinically Relevant Levels of Breast Cancer-Related Single-Nucleotide Variants and Copy Number Variants Using Simulated Cell-Free DNA. J Mol Diagn (2017) 19(4):525–36. 10.1016/j.jmoldx.2017.04.007 28502728

[30] Jia Q Wu W Wang Y Alexander PB Sun C Gong Z. Local mutational diversity drives intratumoral immune heterogeneity in non-small cell lung cancer. Nat Commun (2018) 9(1):5361. 10.1038/s41467-018-07767-w 30560866 PMC6299138

[31] Wang Y Zhao C Chang L Jia R Liu R Zhang Y. Circulating tumor DNA analyses predict progressive disease and indicate trastuzumab-resistant mechanism in advanced gastric cancer. EBioMedicine (2019) 43:261–9. 10.1016/j.ebiom.2019.04.003 PMC6562020 31031019

[32] Niu B Ye K Zhang Q Lu C Xie M McLellan MD. MSIsensor: microsatellite instability detection using paired tumor-normal sequence data. Bioinformatics (2014) 30(7):1015–6. 10.1093/bioinformatics/btt755 PMC3967115 24371154

[33] Szolek A Schubert B Mohr C Sturm M Feldhahn M Kohlbacher O. OptiType: precision HLA typing from next-generation sequencing data. Bioinformatics (2014) 30(23):3310–6. 10.1093/bioinformatics/btu548 PMC4441069 25143287

[34] Szolek A. HLA Typing from Short-Read Sequencing Data with OptiType. Methods Mol Biol (2018) 1802:215–23. 10.1007/978-1-4939-8546-3_15 29858812

[35] Aggarwal C Abreu DR Felip E Carcereny E Gottfried M Wehler T. Prevalence of PD-L1 expression in patients with non-small cell lung cancer screened for enrollment in KEYNOTE-001, -010, and -024 , in ESMO. Ann Oncol (2016) 27(S6):VI363. 10.1093/annonc/mdw378.14

[36] Vanderwalde A Spetzler D Xiao N Gatalica Z Marshall J. Microsatellite instability status determined by next-generation sequencing and compared with PD-L1 and tumor mutational burden in 11,348 patients. Cancer Med (2018) 7(3):746–56. 10.1002/cam4.1372 PMC5852359 29436178

[37] Yarchoan M Albacker LA Hopkins AC Montesion M Murugesan K Vithayathil TT. PD-L1 expression and tumor mutational burden are independent biomarkers in most cancers. JCI Insight (2019) 4(6):e126908. 10.1172/jci.insight.126908 PMC6482991 30895946

[38] Gao S Li N Gao S Xue Q Ying J Wang S. Neoadjuvant PD-1 inhibitor (Sintilimab) in NSCLC. J Thorac Oncol (2020) 15(5):816–26. 10.1016/j.jtho.2020.01.017 32036071

[39] Van Allen EM Miao D Schilling B Shukla SA Blank C Zimmer L. Genomic correlates of response to CTLA-4 blockade in metastatic melanoma. Science (2015) 350(6257):207–11. 10.1126/science.aad0095 PMC5054517 26359337

[40] Rizvi NA Hellmann MD Snyder A Kvistborg P Makarov V Havel JJ. Cancer immunology. Mutational landscape determines sensitivity to PD-1 blockade in non-small cell lung cancer. Science (2015) 348(6230):124–8. 10.1126/science.aaa1348 PMC4993154 25765070

[41] Hellmann MD Ciuleanu TE Pluzanski A Lee JS Otterson GA Audigier-Valette C. Nivolumab plus Ipilimumab in Lung Cancer with a High Tumor Mutational Burden. N Engl J Med (2018) 378(22):2093–104. 10.1056/NEJMoa1801946 PMC7193684 29658845

[42] Ready N Hellmann MD Awad MM Otterson GA Gutierrez M Gainor JF. First-Line Nivolumab Plus Ipilimumab in Advanced Non-Small-Cell Lung Cancer (CheckMate 568): Outcomes by Programmed Death Ligand 1 and Tumor Mutational Burden as Biomarkers. J Clin Oncol (2019) 37(12):992–1000. 10.1200/JCO.18.01042 30785829 PMC6494267

[43] Rizvi NA Cho BC Reinmuth N Lee KH Ahn M-J Luft A. Durvalumab with or without tremelimumab vs platinum-based chemotherapy as first-line treatment for metastatic non-small cell lung cancer: MYSTIC, in ESMO. Ann Oncol (2018) 29(S10):X40–1. 10.1093/annonc/mdy511.005

[44] Velcheti V Kim ES Mekhail T Dakhil C Stella PJ Shen X. Socinski, Prospective clinical evaluation of blood-based tumor mutational burden (bTMB) as a predictive biomarker for atezolizumab (atezo) in 1L non-small cell lung cancer (NSCLC): Interim B-F1RST results., in ASCO. J Clin Oncol (2018) 36(15):S12001. 10.1200/JCO.2018.36.15_suppl.12001

[45] Cristescu R Mogg R Ayers M Albright A Murphy E Yearley J. Pan-tumor genomic biomarkers for PD-1 checkpoint blockade-based immunotherapy. Science (2018) 362(6411):eaar3593. 10.1126/science.aar3593 30309915 PMC6718162

[46] Goodman AM Kato S Bazhenova L Patel SP Frampton GM Miller V. Tumor Mutational Burden as an Independent Predictor of Response to Immunotherapy in Diverse Cancers. Mol Cancer Ther (2017) 16(11):2598–608. 10.1158/1535-7163.MCT-17-0386 PMC5670009 28835386

[47] Yarchoan M Hopkins A Jaffee EM. Tumor Mutational Burden and Response Rate to PD-1 Inhibition. N Engl J Med (2017) 377(25):2500–1. 10.1056/NEJMc1713444 PMC6549688 29262275

[48] Langer C Gadgeel S Borghaei H Patnaik A Powell S Gentzler R. OA04.05 KEYNOTE-021: TMB and Outcomes for Carboplatin and Pemetrexed With or Without Pembrolizumab for Nonsquamous NSCLC, in WCLC. J Thorac Oncol (2019) 14(10):S216. 10.1016/j.jtho.2019.08.426

[49] Garassino M Rodriguez-Abreu D Gadgeel S Esteban E Felip E Speranza G. OA04.06 Evaluation of TMB in KEYNOTE-189: Pembrolizumab Plus Chemotherapy vs Placebo Plus Chemotherapy for Nonsquamous NSCLC. J Thorac Oncol (2019) 14(10):S216–7. 10.1016/j.jtho.2019.08.427

[50] Shao C Li G Huang L Pruitt S Castellanos E Frampton G. Prevalence of High Tumor Mutational Burden and Association With Survival in Patients With Less Common Solid Tumors. JAMA Netw Open (2020) 3(10):e2025109. 10.1001/jamanetworkopen.2020.25109 33119110 PMC7596577

[51] Le DT Durham JN Smith KN Wang H Bartlett BR Aulakh LK. Mismatch repair deficiency predicts response of solid tumors to PD-1 blockade. Science (2017) 357(6349):409–13. 10.1126/science.aan6733 PMC5576142 28596308

[52] Gupta R Sinha S Paul RN. The impact of microsatellite stability status in colorectal cancer. Curr Probl Cancer (2018) 42(6):548–59. 10.1016/j.currproblcancer.2018.06.010 30119911

[53] Cancer Genome Atlas Research N Kandoth C Schultz N Cherniack AD Akbani R Liu Y. Integrated genomic characterization of endometrial carcinoma. Nature (2013) 497(7447):67–73. 10.1038/nature12113 23636398 PMC3704730

[54] Trabucco SE Gowen K Maund SL Sanford E Fabrizio DA Hall MJ. A Novel Next-Generation Sequencing Approach to Detecting Microsatellite Instability and Pan-Tumor Characterization of 1000 Microsatellite Instability-High Cases in 67,000 Patient Samples. J Mol Diagn (2019) 21(6):1053–66. 10.1016/j.jmoldx.2019.06.011 PMC7807551 31445211

[55] Rizvi H Sanchez-Vega F La K Chatila W Jonsson P Halpenny D. Molecular Determinants of Response to Anti-Programmed Cell Death (PD)-1 and Anti-Programmed Death-Ligand 1 (PD-L1) Blockade in Patients With Non-Small-Cell Lung Cancer Profiled With Targeted Next-Generation Sequencing. J Clin Oncol (2018) 36(7):633–41. 10.1200/JCO.2017.75.3384 PMC6075848 29337640

